# Pre-clinical validation of a humanized anti-EGFR variant III chimeric antigen receptor and phase I trial of CART-EGFRvIII in glioblastoma

**DOI:** 10.1186/2051-1426-2-S3-O1

**Published:** 2014-11-06

**Authors:** Laura A Johnson, John Scholler, Takayuki Ohkuri, Akemi Kosaka, Prachi R Patel, Shannon E McGettigan, Arben Nace, Pramod Thekkat, Andreas Loew, Taylor J Chen, Joseph A Fraietta, Avery D Posey, Alina C Boesteanu, Alexandria P Cogdill, Boris Engels, Reshma Singh, Tucker R Ezell, Neeraja Idamakanti, Gabriela Plesa, John Seykora, Hideho Okada, Carl June, Jennifer Brogdon, Marcela Maus

**Affiliations:** 1 Translational Research Program, University of Pennsylvania Perelman School of Medicine, Philadelphia, PA, USA; 2 University of Pennsylvania, Philadelphia, PA, USA; 3 University of Pittsburgh, Pittsburgh, PA, USA; 4 University of Pennsylvania, Hatboro, PA, USA; 5 University of Pennsylvania, Landenberg, PA, USA; 6 Novartis Institutes of BioMedical Research Inc, Quincy, MA, USA; 7 Novartis Institutes for Biomedical Research Inc, Cambridge, MA, USA; 8 University of Pennsylvania Abramson Cancer Center, Wilow Grove, PA, USA; 9 Novartis, Burlington, VT, USA; 10 University of Pennsylvania, Blue Bell, PA, USA; 11 University of California San Francisco, San Francisco, CA, USA; 12 Abramson Cancer Center, Dept. of Medicine, University of Pennsylvania Perelman School of Medicine, Bryn Mawr, PA, USA

## 

Chimeric antigen receptors are synthetic molecules designed to re-direct T cells to specific surface antigens; CAR-modified T cells can mediate long-term durable remissions in B cell malignancies, but expanding this platform to solid tumors requires the discovery of novel surface targets with limited expression. The variant III mutation of the epidermal growth factor receptor (EGFR variant III) is the most common variant of the EGF receptor observed in human tumors, and results from an in-frame deletion of a portion of the extracellular domain. In glioblastoma, the EGFRvIII mutation is oncogenic, portends a poor prognosis, and is thought to be enriched in glioblastoma stem cells. However, because the neoepitope of EGFR variant III is based on a small peptide sequence, an antibody or single-chain variable fragment (scFv) directed to this epitope must be rigorously tested to confirm lack of cross-reactivity to the ubiquitously expressed normal EGFR. Having selected a candidate murine scFv directed to EGFRvIII and a vector backbone encoding a second generation CAR, we generated a panel of humanized scFv's and tested their specificity and function as soluble proteins and in the form of CAR-transduced T cells. The lead candidate scFv was tested in vitro for its ability to direct CAR-transduced T cells to kill antigen-bearing targets effectively, and proliferate and secrete cytokines specifically in response to antigen. We further evaluated the specificity of the lead candidate CAR by comparing it to a cetuximab-based CAR which does not discriminate between EGFR and EGFR variant III; the two CARs, along with negative controls, were tested in vitro against primary cells derived from a panel of normal tissues, and in vivo in immunodeficient mice grafted with normal human skin, which naturally expresses EGFR. CAR-T cells were also able to control tumor growth in xenogeneic subcutaneous and orthotopic models of human EGFR variant III+ glioblastoma. We have designed a Phase I clinical study of CAR T cells transduced with humanized scFv directed to EGFR variant III in patients with glioblastoma.

